# The Burden of Arrhythmias and Conduction Abnormalities Among Patients Presenting With Palpitations at a Cardiac Specialist Care Center in Ghana: A Retrospective Cross‐Sectional Study

**DOI:** 10.1002/hsr2.72732

**Published:** 2026-06-28

**Authors:** Gordon Manu Amponsah, Kwadwo Faka Gyan, Emmanuel Acheamfour‐Akowuah, Philomena Owusu, Isaac Kofi Owusu

**Affiliations:** ^1^ Department of Physiology Kwame Nkrumah University of Science and Technology Kumasi Ghana; ^2^ Directorate of Medicine Komfo Anokye Teaching Hospital Kumasi Ghana; ^3^ Department of Medicine Kwame Nkrumah University of Science and Technology Kumasi Ghana; ^4^ Precise Specialist Clinic Ltd. Kumasi Ghana

**Keywords:** arrhythmias, conduction abnormalities, Holter ECG, Kumasi, palpitations

## Abstract

**Background and Aims:**

Palpitation is one of the commonest presenting complaints to the hospital. The underlying problem may or may not be cardiac. Symptoms may not be present at the time of hospital presentation, and the patient's resting electrocardiogram (ECG) may be normal. In such cases, Holter ECG becomes a valuable tool for evaluating these patients.

**Methodology:**

This 12‐year retrospective cross‐sectional study determined the prevalence of arrhythmias and conduction abnormalities among patients who presented to a specialist cardiac clinic with palpitations and underwent 24‐h Holter ECG monitoring between 2010 and 2022. Data were summarized using descriptive statistics. Also, binary logistic regression models, adjusted for age, sex and comorbidity, were used to examine associations between clinical characteristics and Holter ECG outcomes.

**Results:**

A total of 495 patients were included in the study. Overall, 223 (45.1%) had significant arrhythmia. Frequent ectopic beats were present in 159 (32.0%). Supraventricular ectopic beats and ventricular ectopic beats of any frequency were present in 483 (97.6%) and 402 (81.2%), respectively. Other arrhythmias included non‐sustained ventricular tachycardia (5.5%), atrial fibrillation (1.8%), Mobitz II atrioventricular block (2.2%), sinus pause (> 2 s, 14.9%), and sinoatrial node block/arrest (2.8%). Significant arrhythmias were more likely with advancing age (adjusted odds ratio (aOR) = 11.022, 95% confidence interval (CI): 1.008–1.036, *p* = 0.002), whilst frequent ectopic beats were also more likely with advancing age (aOR 1.017, CI 1.002–1.031, *p* = 0.026) and with the presence of heart failure (aOR = 1.917, 95% CI = 1.048–3.506, *p* = 0.035).

**Conclusion:**

Holter ECG is a valuable tool for evaluating patients with palpitations. Nearly half of the patients showed significant arrhythmia on Holter ECG monitoring.

## 1 Introduction

Palpitation is a very common presenting complaint in the cardiac clinic [1, 2]. It is the most common indication for Holter electrocardiogram (ECG) monitoring [3]. Palpitations may be due to cardiac arrhythmias in about 43% of cases, neuro‐psychiatric causes in 31% (such as anxiety disorders), to other causes in 10% (such as anemia and thyrotoxicosis), and in 16% of cases, the cause of the palpitations cannot be found [4].

A Holter ECG monitor is an ambulatory electrocardiogram (ECG) that provides continuous 24‐h monitoring of cardiac electrical activity. It may be extended to 48–72 h to increase its yield [5]. It is indicated for evaluating palpitations and may detect paroxysmal arrhythmias that are not present on resting ECG. It is also useful for evaluating cardiogenic causes of syncope, assessing response to antiarrhythmic treatment, and predicting the risk of arrhythmia and even mortality [3, 6, 7, 8, 9, 10, 11].

Ectopic beats are the commonest cause of palpitations of cardiac origin [1], although generally, common findings on Holter ECG monitoring include prolonged QT and sinus arrhythmia [3, 12]. Other arrhythmias and conduction disorders found during Holter ECG monitoring include: sinus tachycardia, paroxysmal supraventricular tachycardia, atrial fibrillation, atrial flutter, multifocal atrial tachycardia, ventricular tachycardia, and atrioventricular blocks [1, 3, 4, 12, 13].

Determining the type of arrhythmia is important because some arrhythmias may carry the risk of syncope, reveal underlying coronary artery disease, or provide pointers to the risk of sudden cardiac death (SCD), as occurs in patients with heart failure or cardiomyopathies [8, 9, 10, 11]. Palpitations of cardiac origin may increase the risk of adverse cardiac outcomes and, therefore, should be evaluated with Holter ECG monitoring [14, 15]. There have been many studies globally on the burden of arrhythmias in patients with palpitations. However, little data on this is available in Ghana, particularly in Kumasi.

We sought to determine the burden of arrhythmias and conduction abnormalities among patients with palpitations who underwent Holter ECG monitoring and to assess their relationship to cardiovascular diseases, including hypertension, diabetes mellitus, and heart failure. This research has provided insight into the types of arrhythmias in Ghanaians in Kumasi presenting to the hospital with palpitations and how these arrhythmias correlate with patients' cardiovascular histories. This may guide clinicians in their empirical management of patients with palpitations in Ghana.

## 2 Methodology

### 2.1 Study Design

This study was a 12‐year retrospective cross‐sectional study that employed quantitative techniques.

### 2.2 Study Area

The study was done at the Precise Specialist Clinic Ltd, a private specialist cardiac care center in Kumasi, the capital of the Ashanti Region of Ghana. Kumasi is in southern‐central Ghana and covers 254 km^2^, with an estimated population of about 5 million. Precise Specialist Clinic Ltd. provides specialist general cardiology care and cardiac investigations, including resting ECG, Holter ECG monitoring, resting echocardiogram, stress ECG, ambulatory blood pressure monitoring, and ankle‐brachial index measurement.

### 2.3 Study Population and Study Period

The study population included all adults aged 18 years and above who underwent Holter ECG monitoring for palpitations at Precise Specialist Clinic Limited between 2010 and 2022.

#### 2.3.1 Inclusion Criteria

- Age 18 years and above.
- The primary indication for Holter monitoring was palpitations
- The Holter ECG should have been recorded for a minimum of 18 h and a maximum of 24 h.
- Normal initial resting standard 12‐lead ECG or sinus arrhythmia.
- Available information on participants' age, sex, hypertension, diabetes mellitus, and heart failure, including participants' diary during the Holter ECG monitoring.

#### 2.3.2 Exclusion Criteria

- Holter ECG recorded for less than 18 h or for more than 24 h.
- Holter ECG done for indications other than palpitations.
- Abnormal rhythm on initial resting standard 12‐lead ECG, apart from sinus arrhythmia.
- Information on one or more of the following not available: participants' age, sex, hypertension, diabetes mellitus, heart failure, and participants' diary during the Holter ECG monitoring.

### 2.4 Sample Size and Sampling Technique

Any consecutive Holter ECG recording meeting the inclusion and exclusion criteria was selected for the study. Overall, 495 Holter ECG recordings were included.

### 2.5 Data Collection

The electronic Holter ECG data bank of Precise Specialist Clinic Ltd was manually analyzed, and Holter ECGs meeting the inclusion and exclusion criteria were selected. Each selected case was reviewed and re‐analyzed by two independent cardiologists using the Norav Holter ECG NH301 management system, who provided their independent reports. When their reports differed, the cardiologists re‐analyzed the results together and agreed on a single conclusion. The outcomes were classified into one or more of the following: no arrhythmia or conduction abnormality, sinus tachycardia, sinus arrhythmia, sinus bradycardia, supraventricular/ventricular ectopic beats, atrial fibrillation, atrial flutter, multifocal atrial rhythm, multifocal atrial tachycardia, junctional tachycardia, supraventricular tachycardia, ventricular tachycardia (sustained and non‐sustained), atrioventricular block, sinus pause, or sinoatrial exit block. Information on patients' Holter ECG diary, sex, age, and history of hypertension, diabetes mellitus, and heart failure was also obtained from the medical records.

### 2.6 Definition of Terms Used for This Study

#### 2.6.1 Significant Arrhythmia

The presence of sinus tachycardia, sinus bradycardia, supraventricular ectopic beats (SVE) or ventricular ectopic beats (VE) at least 30 per hour, or the presence of atrial fibrillation, atrial flutter, supraventricular tachycardia, multifocal atrial tachycardia, ventricular tachycardia, ventricular fibrillation, or heart blocks.

#### 2.6.2 Lower‐Risk Holter ECG Findings

Sinus rhythm or sinus arrhythmia (in those under 50 years), with fewer than 30 SVEs or VEs per hour and absence of any other arrhythmia or heart blocks.

#### 2.6.3 No Arrhythmia

Normal sinus rhythm with no significant arrhythmia or lower‐risk Holter findings, as defined above.

#### 2.6.4 Sinus Tachycardia

Sinus rhythm with a heart rate of more than 100 beats per minute (bpm) occurring at rest or during usual daily activities.

#### 2.6.5 Sinus Bradycardia

Sinus rhythm with a heart rate less than 60 bpm during wake periods (6 a.m.–11 p.m.) or less than 40 bpm during sleep periods (11 p.m.–6 a.m.).

Frequent ectopic beats = SVE or VE more than 30/hour or 720/24 h.

### 2.7 Statistical Analysis

Data were entered into Microsoft Excel 2019. Data were cross‐checked to avoid errors such as duplicate entries and then exported to the Statistical Package for the Social Sciences, version 26 (SPSS Inc., United States) for statistical analysis. Descriptive statistics were used to summarize participant characteristics and Holter ECG outcomes. Means and standard deviations were used to describe the continuous variables, and categorical variables were summarized using frequencies and percentages. Differences in continuous and categorical variables were examined using Fisher's exact test and Pearson's *χ* ^2^ test. Binary logistic regression models, adjusted for age, sex, hypertension, diabetes mellitus, and heart failure, were used to examine associations between clinical characteristics and Holter ECG outcomes. Multicollinearity was assessed using a correlation matrix, which indicated no multicollinearity. An omnibus test of model coefficients was conducted to evaluate overall model fit. An alpha value of *p* < 0.05 was considered statistically significant in all analyses.

### 2.8 Ethical Consideration

Ethical approval (CHRPE/AP/081/23) was obtained from the Committee on Human Rights and Publication Ethics at Kwame Nkrumah University of Science and Technology, Kumasi, Ghana. The data obtained were analyzed solely for the purposes of the study, and the utmost discretion was exercised in handling patients' personal information.

## 3 Results

### 3.1 Baseline Clinical Characteristics of Participants

Four hundred and ninety‐five (495) patient records were analyzed. The proportions of males and females were almost equal, with 241 (48.7%) and 254 (51.3%), respectively. The mean age was 53 ± 16 years, and 180 (36.4%) of the participants were 60 years or older. More than half, 287 (58.0%), of participants had at least one cardiovascular comorbidity, as shown in Table 1.

**Table 1:** Clinical characteristics of overall participants and those with significant arrhythmia.

Characteristics	Overall *n* (%)	Significant arrhythmias *n* (%)	*χ* ^2^	*p* value
**Total participants**	495 (100.0)	223 (45.1)	—	—
**Sex**				
**Male**	241 (48.7)	108 (44.8)	0.01	0.91
**Female**	254 (51.3)	115 (45.3)		
**Age,** mean ± SD (years) = 53 ± 16				
Age, categories				
≤ 40	128 (25.9)	53 (23.8)	23.47	< 0.001
41–60	187 (37.8)	64 (28.7)		
> 60	180 (36.4)	106 (47.5)		
**Comorbidities**				
**Hypertension**	259 (52.3)	84 (37.7)	0.36	0.548
**Diabetes mellitus**	67 (13.5)	91 (40.8)	2.36	0.125
**Heart failure**	55 (11.1)	45 (20.2)	7.03	0.008
**Presence of at least one comorbidity**	287 (58.0)	139 (62.3)	3.16	0.076
**Number of comorbidities**				
0	208 (42.0)	84 (37.7)	4.81	0.186
1	198 (40.0)	91 (40.8)		
2	84 (17.0)	45 (20.2)		
3	5 (1.0)	3 (1.3)		

Abbreviation: SD = standard deviation.

Baseline rhythm was sinus in all participants, with 31 (6.3%) exhibiting baseline sinus arrhythmia. All participants had at least one form of arrhythmia, with 223 (45.1%) having significant arrhythmias and 274 (55.9%) with lower‐risk Holter findings (Table 2).

**Table 2:** Frequency of Holter monitoring outcomes (*n* = 495).

Event	Frequency *n* (%)
No arrhythmia	0 (0)
Lower‐risk Holter findings	274 (55.9)
Significant arrhythmia	223 (45.1)
Sinus rhythm	464 (93.7)
Sinus arrhythmia	31 (6.3)
Sinus tachycardia	243 (49.1)
Sinus bradycardia	86 (17.4)
Atrial fibrillation	9 (1.8)
Atrial flutter	2 (0.4)
Multifocal atrial tachycardia	1 (0.2)
Junctional rhythm	0
Supraventricular ectopic beats	483 (97.6)
Supraventricular ectopic beats burden > 5%	47 (9.5)
Paroxysmal supraventricular tachycardia	24 (4.8)
Ventricular ectopic beats	402 (81.2)
Ventricular ectopic beats burden > 5%	30 (6.1)
Non‐sustained ventricular tachycardia	27 (5.5)
Sustained ventricular tachycardia	0
First‐degree AV block	3 (0.6)
Mobitz 1 AV block	1 (0.2)
Mobitz 2 AV block	11 (2.2)
Complete heart block	0
Sinus pause > 2 s	74 (14.9)
Sinoatrial block/arrest	14 (2.8)
Frequent ectopic beats	159 (32.1)

Abbreviation: AV = atrioventricular.

### 3.2 Prevalence of Arrhythmias

Table 2 summarizes the arrhythmias recorded. Although presented individually, some occurred simultaneously in the same patient. The most common arrhythmia was SVE, present in 483 (97.6%), followed by VE, 402 (81.2%). However, only 47 (9.5%) and 30 (6.1%) of patients had SVE and VE greater than 5%, respectively. Overall, 159 (32.1%) had frequent ectopic beats, with paroxysmal supraventricular tachycardia in 24 (4.8%) and non‐sustained ventricular tachycardia (NSVT) in 27 (5.5%), as shown in Table 2. Paroxysmal atrial fibrillation occurred in 9 (1.8%) of patients (Table 2). Although female sex appeared protective, as shown in Table 4, the multivariable binary logistic regression model for atrial fibrillation lacked stability due to the rarity of events (an omnibus test of model coefficients *p* value = 0.068; Nagelkerke R‐squared = 0.124). Advancing age and heart failure were significantly associated with the development of significant arrhythmia (*p *< 0.001 and *p*= 0.008, respectively) as shown in Table 1.

### 3.3 Burden of Ectopic Beats

Among patients who developed VE, 30 (6.1%) had a VE burden above 5% (Table 2). As shown in Table 3, the frequency of VE was associated with age (*p* = 0.037), hypertension (*p* = 0.071), and having more comorbidities (*p* = 0.013). However, no significant predictors were found on multivariate analysis.

**Table 3:** Clinical characteristics of participants according to ventricular ectopic beats and supraventricular ectopic beats burden.

Characteristics	Total (%)	< 1%	1%–5%	5.1%–10%	> 10%	*p* value
Ventricular ectopic beats						
Overall	495 (100)	428 (86.5)	37 (7.5)	15 (3)	15 (3)	
Male	241 (48.7)	216 (85)	21 (8.3)	8 (3.1)	9 (3.5)	0.72
Female	254 (51.3)	212 (88)	16 (6.6)	7 (2.9)	6 (2.5)	
Age category (years)						
≤ 40	128 (25.9)	119 (93)	4 (3.1)	2 (1.6)	3 (2.3)	0.037
41–60	187 (37.8)	163 (87.2)	13 (7)	8 (4.3)	3 (1.6)	
> 60	180 (36.4)	146 (81.1)	20 (11.1)	5 (2.8)	9 (5)	
Comorbidity	287 (58)	220 (76.7)	33 (11.5)	14 (4.9)	20 (7)	0.071
Hypertension	259 (52.3)	214 (82.6)	28 (10.8)	8 (3.1)	9 (3.5)	0.025
Diabetes mellitus	67 (13.5)	56 (83.6)	7 (10.4)	3 (4.5)	1 (1.5)	0.54
Heart failure	55 (11.1)	43 (78.2)	7 (12.7)	2 (3.6)	3 (5.5)	0.26
Number of comorbidities						
0	208 (42)	195 (93.8)	5 (2.4)	4 (1.9)	4 (1.9)	0.013
1	198 (40)	157 (79.3)	23 (11.6)	9 (4.5)	9 (4.5)	
2	84 (17)	72 (85.7)	8 (9.5)	2 (2.4)	2 (2.4)	
3	5 (1)	4 (80)	1 (20)	0	0	
Supraventricular ectopic beats						
Overall	495 (100)	390 (78.8)	58 (11.7)	23 (4.6)	24 (4.8)	
Male	241 (48.7)	187 (77.6)	32 (13.3)	8 (3.3)	14 (5.8)	0.29
Female	254 (51.3)	203 (79.9)	26 (10.2)	15 (5.9)	10 (3.9)	
Age category (years)						
≤ 40	128 (25.9)	102 (79.7)	20 (15.6)	6 (4.7)	0	< 0.001
41–60	187 (37.8)	164 (87.7)	14 (7.5)	4 (2.1)	5 (2.7)	
> 60	180 (36.4)	124 (68.9)	24 (13.3)	13 (7.2)	19 (10.6)	
Comorbidity	287 (58)	220 (76.7)	33 (11.5)	14 (4.9)	20 (7)	0.081
Hypertension	259 (52.3)	204 (78.8)	26 (10)	12 (4.6)	17 (6.6)	0.21
Diabetes mellitus	67 (13.5)	51 (76.1)	9 (13.4)	3 (4.5)	4 (6)	0.92
Heart failure	55 (11.1)	33 (60)	11 (20)	3 (5.5)	8 (14.5)	< 0.001
Number of comorbidities						
0	208 (42)	170 (81.7)	25 (12)	9 (4.3)	4 (1.9)	0.13
1	198 (40)	154 (77.8)	22 (11.1)	10 (5.1)	12 (6.1)	
2	84 (17)	64 (76.2)	9 (10.7)	4 (4.8)	7 (8.3)	
3	5 (1)	2 (40)	2 (40)	0	1 (20)	

The burden of SVE above 5% was observed in 47 (9.5%) participants (Table 2) and was significantly associated with age (*p *< 0.001) and a history of heart failure (*p *< 0.001), as shown in Table 3. In multivariate analysis, it remained significantly associated with age (aOR 1.051, 95% CI (1.026–1.076), *p* < 0.001

Those with advancing age (aOR = 1.017, 95% CI = 1.002–1.031, *p*= 0.026) and those with a history of heart failure (aOR = 1.917, 95% CI = 1.048–3.506, *p*= 0.035) were more likely to develop frequent ectopic beats, as shown in Table 4.

**Table 4:** Fully adjusted binary logistic regression models exploring the relationship between Holter monitoring outcomes and clinical characteristics of all participants.

Characteristics	Significant arrhythmias	Frequent ectopic beats	SVE burden > 5%	VE burden > 5%	Atrial fibrillation
Age	1.022	1.017	1.051	1.024	1.032
	(1.008–1.036)	(1.002–1.031)	(1.026–1.076)	(0.996–1.052)	(0.979–1.089)
	0.002	0.026	< 0.001	0.094	0.243
Sex, female	1.049	0.924	1.095	1.289	0.105
	(0.730–1.509)	(0.629–1.359)	(0.585–2.048)	(0.608–2.734)	(0.013–0.861)
	0.795	0.688	0.777	0.508	0.036
Hypertension	0.743	0.906	0.808	0.870	1.345
	(0.488–1.131)	(0.582–1.409)	(0.400–1.629)	(0.373–2.030)	(0.292–6.188)
	0.166	0.661	0.550	0.747	0.704
Diabetes mellitus	1.296	1.052	0.754	0.786	1.402
	(0.745–2.252)	(0.592–1.872)	(0.307–1.852)	(0.253–2.445)	(0.248–7.917)
	0.359	0.862	0.538	0.677	0.702
Heart failure	1.589	1.917	1.392	1.158	1.632
	(0.861–2.932)	(1.048–3.506)	(0.612–3.165)	(0.390–3.443)	(0.298–8.923)
	0.139	0.035	0.430	0.791	0.572

*Note:* Data are presented as adjusted odds ratio (95% confidence interval), *p* value. *n* = 495.

Abbreviations: SVE = supraventricular ectopic beats, VE = ventricular ectopic beat.

## 4 Discussion

In this study, we report the burden of arrhythmias among patients evaluated with 24‐h Holter ECG monitoring for palpitations in Kumasi, Ghana. Significant arrhythmias were identified in nearly half of the patients (45.1%). In keeping with our findings, Weber and Kapoor found that 43% of patients presenting with palpitations had arrhythmias [4]. A study by Chiang also reported a similar prevalence of around 39.1% [12].

The most common arrhythmia subtype in our study was ectopic beats (SVE and VE), with 32% of patients having frequent ectopic beats. This finding aligns with an earlier study by Paudel and Paudel, which reported a 40.3% prevalence of frequent ectopic beats (> 30/hour) [6]. The prevalence of VE burden above 5% was 6.1%, similar to the finding of Torrado et al., who reported a prevalence of 4% [16].

Beyond the high frequency of ectopic beats, more complex rhythm disturbances observed in this cohort, including NSVT (5.5%), atrial fibrillation (1.8%), Mobitz II atrioventricular block (2.2%), sinus pause (14.9%), and sinoatrial node (SAN) block/arrest (2.8%), are clinically important. Detecting NSVT is important because of the risk of SCD. Studies demonstrate that NSVT correlates with an elevated risk of SCD in individuals with pre‐existing structural heart disease [15] and even in those without structural heart disease [17]. In addition to its prognostic significance, NSVT provides a diagnostic window into cardiac health; it often indicates subclinical structural heart disease, myocardial ischemia, or electrolyte imbalances that may not be detectable on a standard resting ECG [18].

Paroxysmal atrial fibrillation was present in 1.8% of patients in this study, similar to the prevalence reported by Karregat et al. (1.5%) [19] and to that of a Portuguese study of 4843 individuals undergoing Holter monitoring for various indications (2.5%) [20]. Although the diagnostic yield of Holter ECG for atrial fibrillation is low, detection remains important, given the increased risk of stroke in this population [20].

High‐degree AV block, sinus pauses, and SAN block/arrest provide more conclusive evidence of substantial conduction system pathology that could progress to symptomatic bradycardia or higher‐grade blocks. They may warrant subsequent investigation and, at times, intervention, including pacemaker insertion [21]. Identifying these specific rhythms through Holter monitoring is more clinically actionable, as it directly identifies patients who need aggressive diagnostic evaluation and therapeutic management to avoid fatal outcomes.

This study also showed that the risk of developing significant arrhythmias in general, and of frequent ectopic beats, increased with advancing age. This is consistent with the literature [22]. Heart failure and systemic hypertension were important associations with the development of significant arrhythmias and with a high VE burden, respectively, which is consistent with the literature [23, 24].

Our study should be interpreted in the context of its design. First, although our study was based on a relatively large retrospective cohort, the findings may not be generalizable to all populations owing to the enrollment method and geographic specificity. Also, it is evident that this report is not a population‐based prevalence study and that it has all the limitations of studies focusing on a single center. With the study site being a private specialist referral center, there is the risk of referral bias, which also limits generalizability. Nonetheless, our findings provide contemporary data on the real‐life situation in this population in Kumasi.

## 5 Conclusion

Holter ECG monitoring is a valuable tool for evaluating patients presenting to the hospital with palpitations; nearly half exhibited significant arrhythmias, with a high prevalence of ectopy and a substantial proportion of clinically defined arrhythmias in this referred population. The use of Holter ECG to evaluate patients with palpitations or suspected paroxysmal arrhythmias should be strongly encouraged to aid arrhythmia diagnosis and guide timely clinical management, thereby improving cardiovascular outcomes in the region.

## Author Contributions

**Gordon Manu Amponsah:** conceptualization, data curation, methodology, formal analysis, writing – original draft, writing – review and editing. **Kwadwo Faka Gyan:** methodology, formal analysis, data curation, writing – review and editing, writing – original draft. **Emmanuel Acheamfour‐Akowuah:** conceptualization, data curation, methodology, formal analysis, writing – original draft. **Philomena Owusu:** visualization, writing – review and editing. **Isaac Kofi Owusu:** conceptualization, data curation, investigation, methodology, supervision, writing – review and editing, writing – original draft, formal analysis.

## Funding

The authors have nothing to report.

## Conflicts of Interest

The authors declare no conflicts of interest.

## Transparency Statement

The corresponding author, Isaac Kofi Owusu, affirms that this manuscript is an honest, accurate, and transparent account of the study being reported; that no important aspects of the study have been omitted; and that any discrepancies from the study as planned (and, if relevant, registered) have been explained.

## Data Availability

The datasets used and/or analyzed during the current study are available from the corresponding author on reasonable request.
